# Strained Silicon Photonics

**DOI:** 10.3390/ma5050889

**Published:** 2012-05-22

**Authors:** Clemens Schriever, Christian Bohley, Jörg Schilling, Ralf B. Wehrspohn

**Affiliations:** 1 Centre for Innovation Competence SiLi-nano, Martin-Luther-University Halle-Wittenberg, Karl-Freiherr-von-Fritsch-Str. 3, Halle (Saale) 06120, Germany; E-Mails: christian.bohley@physik.uni-halle.de (C.B.); joerg.schilling@physik.uni-halle.de (J.S.); 2 Fraunhofer Institute for Mechanics of Materials, Walter-Hülse-Str. 1, Halle (Saale) 06120, Germany; E-Mail: ralf.wehrspohn@iwmh.fraunhofer.de; 3 Institute of Physics, Martin-Luther-University Halle-Wittenberg, Heinrich-Damerow-Str. 4, Halle (Saale) 06120, Germany

**Keywords:** silicon photonics, strain engineering, nonlinear optical properties

## Abstract

A review of recent progress in the field of strained silicon photonics is presented. The application of strain to waveguide and photonic crystal structures can be used to alter the linear and nonlinear optical properties of these devices. Here, methods for the fabrication of strained devices are summarized and recent examples of linear and nonlinear optical devices are discussed. Furthermore, the relation between strain and the enhancement of the second order nonlinear susceptibility is investigated, which may enable the construction of optically active photonic devices made of silicon.

## 1. Introduction

The influence of strain on the mechanical properties of microtechnological devices is a long known effect. Especially the miniaturization of microelectronic components has led to great efforts in investigating the sources of strain, as this was often a reason for device degradation. The strains originate from different processing steps in the CMOS processing line where materials are brought into contact at high temperatures. The difference in the thermal expansion coefficients can then lead to stresses between the materials. This problem becomes even more significant, when the miniaturization leads to devices on the nanoscale, with dimensions equal to the thicknesses of the strained layers. However, besides the undesired effects like delamination, cracking and device degradation, strain can also be used to enhance the desired physical properties of nanostructured devices, like it is already done in the field of microelectromechanical systems (MEMS) or strained silicon CMOS technology (SS CMOS). Especially the latter gives a good example how strain can be used to improve the electrical properties of silicon. Here, the lattice structure of silicon is stretched by bonding it onto a SiGe substrate [1]. The larger lattice constant leads to a tensile strain in the silicon and thus to a distortion of the electronic band structure. This in turn leads to an enhancement of the electron mobility by up to 70%. Today strained silicon electronics has become a mature technology and is now commonly used for the fabrication of microelectronic devices.

Another field of work that is still developing is the field of strained silicon photonics. Here, strain is used to modify and enhance the spectrum of linear and nonlinear optical properties of photonic devices. In this paper the progress that has been made in this field is reviewed. It starts with a summary of technologies that can be used to create strains in optical silicon devices (Section 2). Further, the application of strain for improving integrated linear optical structures is discussed in Section 3.

Another promising field of work is the use of strained silicon for nonlinear optical applications. Due to symmetry reasons, silicon is lacking second order nonlinear properties that are essential for effects like sum- or difference frequency generation or electro-optical modulation based on the Pockels effect. In Section 4 the relation between strain, structural symmetry and second order nonlinear susceptibility is discussed. The paper is concluded by a review of recently published reports on active optical devices that exploit the strain induced second order nonlinearities (Section 5).

## 2. Methods for Creating Strained Silicon

**Figure 1:**
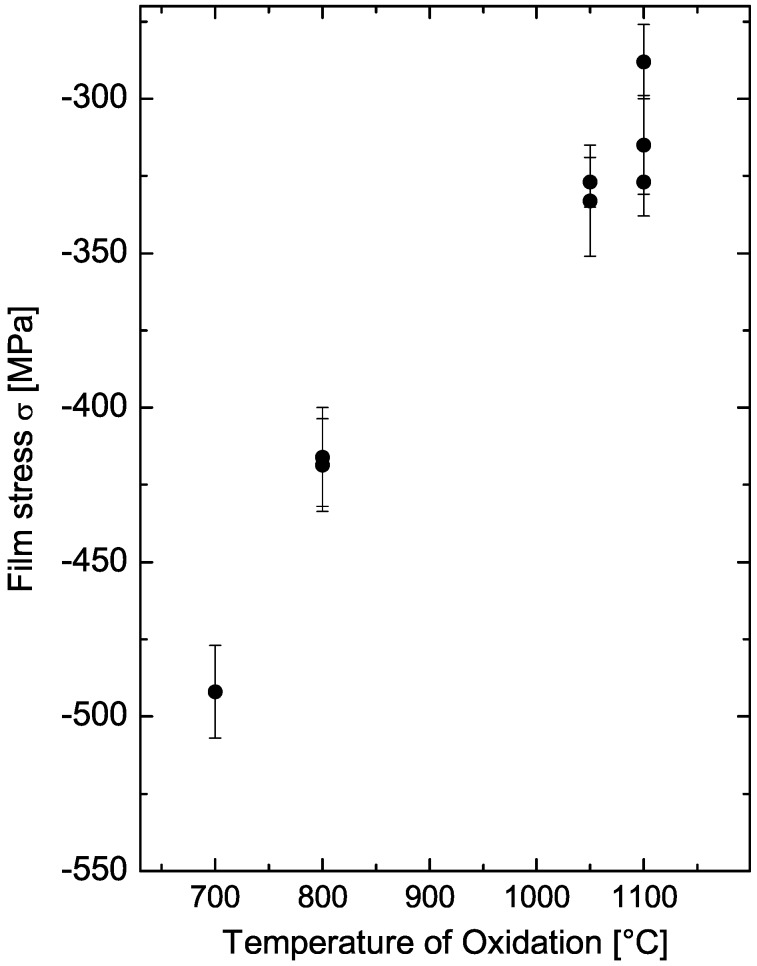
Dependence of silicon oxide film stress on the process temperature. The temperature affects the magnitude of the intrinsic and thermal stress component leading to a variation of the total stress level.

To create the strains needed to alter the optical properties of photonic devices, usually two methods are employed covering the device with a straining layer. A direct mechanical and indirect optomechanical method are described in Section 3. Probably, the simplest method is the use of thermal oxidation to create a straining cover layer on top of a device. With this method a silicon oxide layer is created at high temperatures, whereas the growth rate of the oxide is determined by the process temperature [2]. Further, the process temperature determines the amount of stress that is generated in the oxide layer, at which a lower temperature leads to a higher stress level (see Figure 1).

Usually process temperatures above 700 °C are used, as lower temperatures lead to unreasonably long oxidation times. To achieve higher oxidation rates, the process of wet oxidation, where H_2_O vapor is used instead of pure oxygen, is preferred due to the faster diffusion of the H_2_O molecules into the silicon [3]. The higher growth rate also leads to higher stresses in the oxide layer [4,5].

The stress *σ* that is created by this process originates from two different sources, its respective magnitude depending on the process temperature:

(1) *σ* = *σ*_*th*_ + *σ*_*i*_*.*

Here, *σ*_*th*_ corresponds to the thermal stress created by the mismatch Δ*α* of the thermal expansion coefficients of silicon and silicon oxide. It leads to an unequal contraction of both materials upon cooling down from process temperature to room temperature. Therefore, this component of stress increases with temperature and leads to a compressive stress in the oxide layer because *α*_*Si*_* > α*_*SiO*_2__ . The magnitude of *σ*_*th*_ can be calculated by [6] as

(2) $$\sigma_{th}=\frac{E}{1-\nu }\Delta \alpha \Delta T,$$

where *E* is the Young modulus and *ν* is the Poisson ratio.

The second component of Equation 1 corresponds to the intrinsic stress *σ*_*i*_ of the oxide layer that is created by the volumetric expansion of the oxide during the oxidation process leading to a compressive stress. It originates from the difference in molar volume between silicon and silicon oxide, which is 120% larger than the first. However, this component of strain can only be observed for temperatures below approximately 960 °C [7]. For higher temperatures the viscosity of the grown oxide is too low and leads to a relaxation of the expansion induced strain [8]. For lower temperatures the viscosity increases and thus the stress induced into the oxide layer.

More demanding from a technological point of view is the creation of strains by deposition of a strained silicon nitride layer (SiN) by means of plasma enhanced chemical vapor deposition (PECVD). Here, the cover layer is deposited from a gas plasma consisting of silane (SiH_4_), ammonia (NH_3_) and nitrogen (N_2_). In comparison with thermal oxidation this process allows to create compressive as well as tensile stresses in a broader range. Further, it offers a better control of the stress level to be applied to the device.

The parameters having the strongest influence on the generated stress are the process temperature *T* and the plasma frequency *f*, at which the latter is more suitable as a change in temperature may cause additional unwanted changes in the chemical composition of the film [9]. Similar to the process of thermal oxidation the stress in the SiN layer can be separated into a thermal component *σ*_*th*_ caused by the difference between deposition and room temperature and an intrinsic component induced during the deposition process. In contrast to the thermal oxidation, here *σ*_*th*_ is tensile because Δ*α* is negative in Equation 2 due to the fact that *α*_*Si*_ *< α*_*SiN*_. The component of intrinsic stress *σ*_*i*_, which is dominating the overall stress *σ*, is controlled both by the frequency of the plasma and the process temperature. Depending on the parameters *σ*_*i*_ can be tensile or compressive. Compressive stresses occur for low temperatures and low radio frequencies. The reason is that, for low frequencies which are usually in the range of a few 100 kHz, the ions in the plasma are accelerated by the alternating electric field leading to a bombardment of the growing SiN layer. This causes an expansion of the layer due to implanted atoms and broken Si-N bonds [10,11]. For higher temperatures this effect is reduced, as the implantation damage is annealed out.

For higher radio frequencies the effect of implantation damage can be neglected, as the ions are too slow to be accelerated by the electric field. Here the thermally controlled rate of hydrogen (H_2_) desorption determines the stress produced. In general in the deposition process the H_2_ desorption rate is lower than the rate at which radicals are formed in the plasma and inserted into Si-H and N-H bonds. This creates a SiN layer with high H_2_ concentration. For high temperatures H_2_ desorption and cross-linking continue after the actual deposition step, leading to a shrinkage of the produced film and thus to a tensile intrinsic stress which adds up to the tensile stress of the thermal component [10].

**Figure 2:**
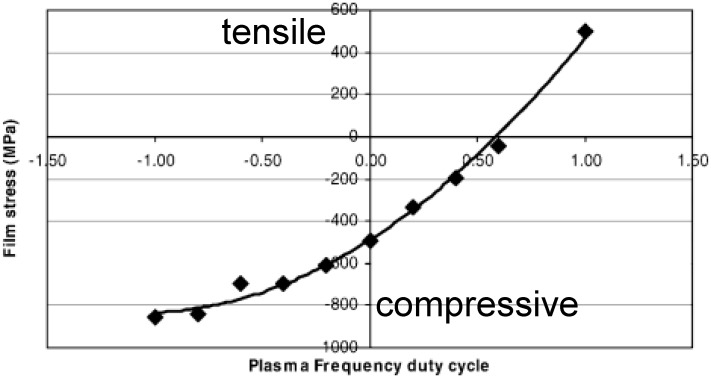
Dependence of plasma deposited silicon nitride film stress on the plasma frequency duty cycle Φ. Reproduced from Reference [12] with courtesy of Wiley InterScience.

A method that is particularly suitable for the creation of strained silicon layer is the Dual-frequency PECVD [9,12,13]. It works with two frequencies, one at 13.56 MHz the other between 50 kHz and 500 kHz. Here, the stress in the nitride layer can be selected by changing the duty cycle Φ of the frequencies (see Figure 2):

(3) $$\Phi =\frac{t_{HF}-t_{LF}}{t_{HF}+t_{LF}}$$

The parameters *t*_*HF*_ and *t*_*LF*_ represent the duration of high and low frequency plasma excitation, respectively in one deposition cycle (*t*_*HF*_ + *t*_*LF*_). In this way layers with stress levels between *−*1000 and +1000 MPa can be generated [12,14].

## 3. Strained Silicon Linear Optical Devices

The strain generated inside silicon by the aforementioned processes can be used for example to induce refractive index changes in waveguiding structures. The possibility of controlling the refractive index is essential for some optical applications. For instance, birefringence tunes the phase-matching of a waveguide and the refractive index of a material controls its optical resonance characteristics. Generally, strain changes the refractive properties of optical materials. Strain in silicon for optically linear effects was induced mechanically [15] or piezoelectrically [16,17,18,19]. The photoelastic effect involved here is the influence of strains on the refractive index tensor in a birefringent medium.

Strain leads to a linear dependence of the refractive index components on the single strain tensor components [20]:

(4) $$n_{ij}(\sigma_{kl})=n_{ij}(0)+\underset{kl}{\sum }C_{ijkl}\sigma_{kl}.$$

The photoelastic tensor *C*_*ijkl*_ depends on structural symmetries of the medium and on the applied strain field. Photoelastic effects are caused by the applied strain since the changed lattice spacing influences the refractive index. Thus, the medium becomes anisotropic, therefore having a nonzero birefringence.

**Figure 3:**
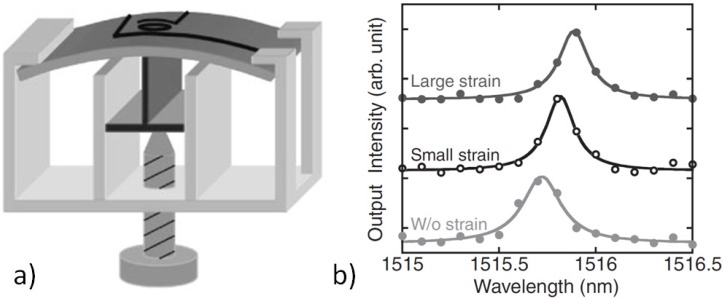
(**a**) Si race track ring resonator on silica bent in a sample holder; (**b**) Resonance characteristics at about 1515.5 nm for horizontal strain. Figure is taken from Reference [15] with courtesy of the Japan Society of Applied Physics.

The photoelastic effect in bulk silicon was reported by Biegelsen [21]. Amemiya *et al.* showed that this effect can be applied in miniature optical devices, here for a silicon race track resonator on an SOI wafer [15]. The ring resonator is bent with a sample holder that induces strain in the whole wafer (see Figure 3). The observed resonance shift is in the order of 0.1 nm. That confirmed the order of the photoelastic constant determined by Biegelsen [21].

**Figure 4:**
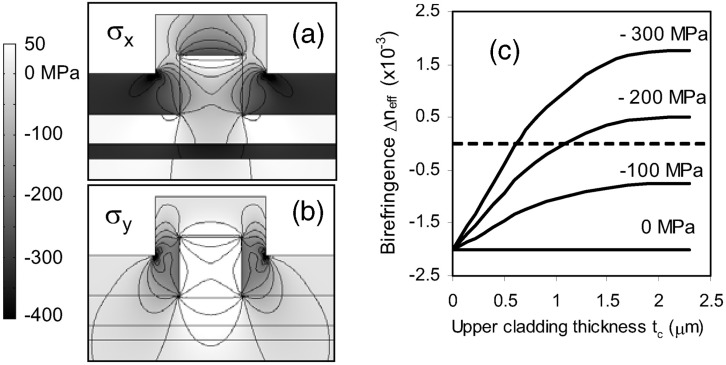
Calculated stress distribution in an SOI waveguide in (**a**) x direction and (**b**) y direction; (**c**) shows the stress and thickness dependence of the birefringence. It saturates at a thickness of 2.5 *µm* because the stress in a thicker layer does not reach the waveguide any more. The figure is taken from [16] with courtesy of the Optical Society of America.

A technologically more relevant and effective method for straining silicon is cladding the waveguiding material. Xu *et al.* [16,17] induced birefringence in a ridge waveguide by the deposition of a straining layer. The birefringence originates from asymmetric conditions of the surrounding material (e.g., air on top, silicon oxide at the bottom) and from geometric deviations in the production process. Biaxial strain in deposited layers generates also strains in the core of the waveguide structure in *x* and *y* direction (Figure 4 (a,b)). Xu *et al.* calculated significant core stress contributions of *σ*_*x*_ *≈ −*70 MPa and *σ*_*y*_ *≈* 180 MPa for a film with a thickness of 370 nm and a stress level of *σ* = *−*320 MPa. The birefringence originating from the geometry is compensated by this stress anisotropy. Beside the tuning of the core stress by changing the external stress level, also the effect of increasing the cladding thickness was reported. This effect enhances the birefringence due to additional core stress (Figure 4c). The film stress is strongly sensitive to deposition conditions. Thus, the variation of the cladding thickness can be suitable for tuning the birefringence. The effect on the refractive index is significant as for a 2 *µ*m thick oxide layer with *σ*_*film*_ = *−*300 MPa an index change of Δ*n* = 1*.*6 *×* 10^*−*3^ is calculated.

**Figure 5:**
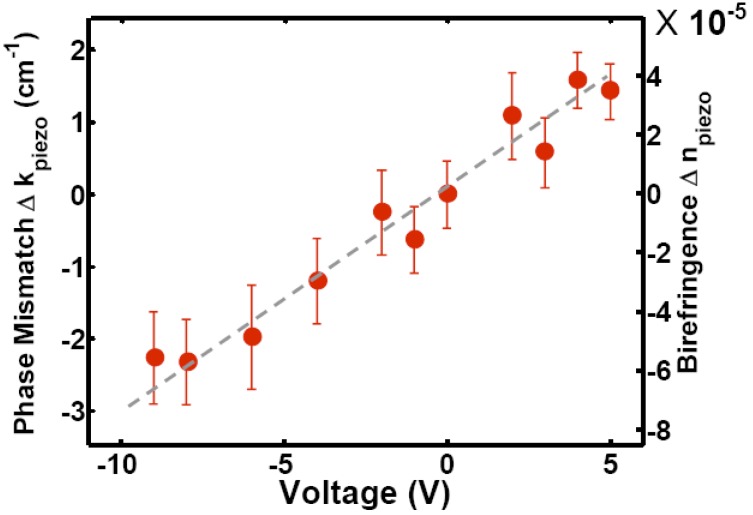
Dependence of phase-mismatch and birefringence of an SOI waveguide on the voltage applied to the piezoelectric manipulator. The figure is taken from [19] with courtesy of the Optical Society of America.

An analog approach is used by Tsia *et al.* [18] bringing the idea a step further by creating a waveguide with electrical tunable birefringence. A ridge waveguides is covered with a transparent layer of 500 nm silicon oxide to avoid absorption losses by the piezoelectric manipulator that is put on top. Varying the applied voltage changes the strain on the layer (Figure 5). The induced stress is much smaller than the stress from the cladding layer, the calculated core stress level induced solely by the piezo is $\sigma_{x}^{piezo}=-1$ MPa and $\sigma_{y}^{piezo}=12$ MPa, respectively. The induced birefringence is Δ*n* *≈* 3 *×* 10^−4^.

In Reference [19], the electrical tunability of the refractive index is used to achieve phase-matching between different light waves in a coherent anti-Stokes Raman scattering (CARS) experiment. The conversion efficiency is enhanced by 5–6 dB if the voltage applied to the piezoelectric device is varied.

Indirectly, strained materials are used also for optomechanical effects [22,23] where optical interaction between the waveguides changes the geometry of the device. Ma *et al.* presented a method for using optical forces between parallel silicon strip waveguides, resulting in a strain that controls the phase and group birefringence [22]. The two strip waveguides of a length of 30 *µ*m that are suspended at the ends have a distance of about 100 nm. For an incident power of 20 mW, optical forces between the waveguides induce a displacement of 9 nm for each strip at the waveguide centers. The device can be used for polarization conversion, whereas a potential application for this idea is in using optical forces in microring resonator systems. That was realized for silicon nitride microresonators leading to resonance shifts in the order of 2 nm [23].

## 4. The Effect of Strain on Second Order Nonlinear Optical Processes in Silicon

Next to the possibility of modifying the linear optical properties of silicon devices by the application of strain, the strain can also be used to alter the nonlinear optical properties of such devices. This is of high interest because ordinary silicon shows only weak nonlinear optical properties. Especially the absence of the second order nonlinear susceptibility *χ*^(2)^ in bulk silicon has hindered the creation of optically active devices relying on second order nonlinear effects like the Pockels effect.

**Figure 6:**
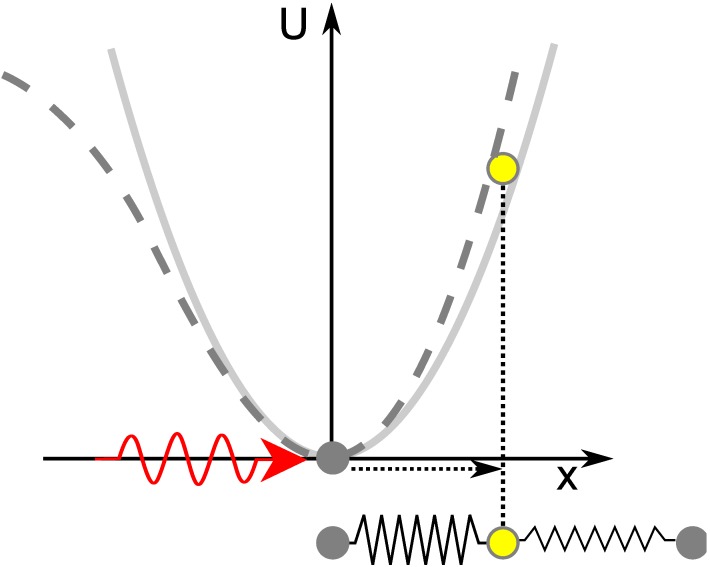
The excitation of an electron (yellow) by a photon (red) can be illustrated by an oscillation in a harmonic potential (solid line). In the case of nonlinear excitation neighboring atoms have to be taken into account as well, leading to asymmetric potentials (dashed) in the case of a non-centrosymmetric medium.

The reason for this can be found in the structural symmetry of the silicon crystal. This can be envisioned with the help of Figure 6 that presents a schematic sketch of linear and second order nonlinear light matter interaction. Unlike in linear optics, where the excitation of an electron can be regarded as an oscillation in a harmonic potential, in nonlinear optics, where high excitation intensities are used, additional asymmetric contributions to the electronic potentials have to be taken into account as well. This can be considered by adding higher order terms to the potential energy function: (5)
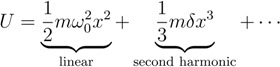


The cubic term responsible for second order nonlinear contributions leads to an asymmetric potential (Figure 6) only existing in non-centrosymmetric media. For centrosymmetric media *δ* vanishes in Equation 5. As silicon is a face centered cubic material with equal atoms, it cannot meet the symmetry conditions required for second order nonlinear processes [24].

The application of strain to the silicon crystal can lead to a qualitative change of the crystal structure. The idea behind this concept is that an *inhomogeneous* strain will break the cubic crystalline symmetry of silicon. That leads to local crystal configurations allowing the existence of asymmetric electronic potentials as described in Equation 5. This in turn gives rise to a dipolar *χ*^(2)^ in the strained bulk crystal.

Experimentally, this can be investigated with the method of second harmonic generation (SHG) where light (*E*^*ω*^) at frequency *ω* is converted into light (*E*^2*ω*^) at the second harmonic frequency 2*ω*: (6)
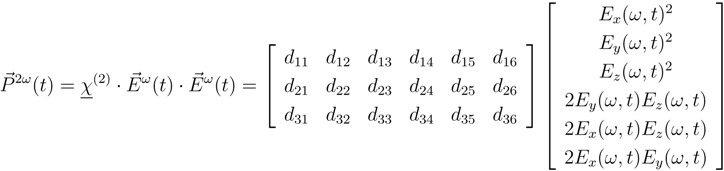


Here, the *d*_*il*_ represent the tensorial components of *χ*^(2)^ written in the contracted Kleinman notation (see Reference [25] for details).

**Figure 7:**
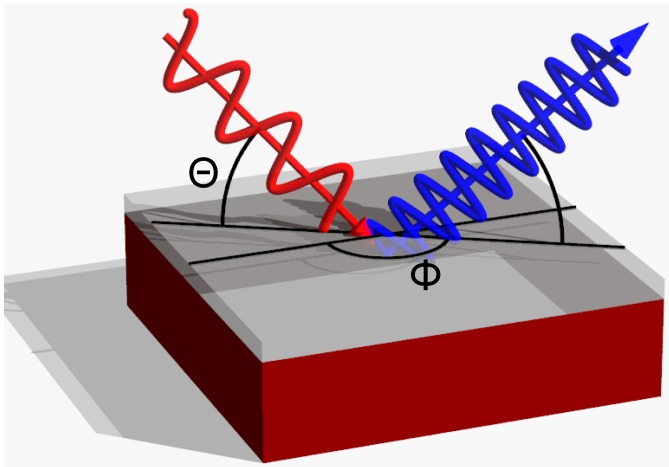
Experimental setup for measuring the second harmonic signal in reflection. The fundamental beam (red) irradiates the sample at an angle Θ, the SH beam (blue) is detected at the opposite angle. By rotating the sample azimuthally by the angle *ϕ* the influence of the lattice structure on the SH signal can be observed.

A common method to observe SHG in strained and unstrained silicon is the measurement of the SH signal in reflection (see Figure 7). Even though a dipolar second order nonlinear susceptibility is forbidden in unstrained bulk silicon, there are different other sources that lead to the generation of a second harmonic signal [26,27]. Generally, these sources can be separated into two parts. One part originates from the interface between the silicon and the adjacent medium. Here the centrosymmetry is locally broken due to the termination of the crystal leading to a dipolar second harmonic polarization:

(7) $$P^{s,dp}(2\omega )=\chi_{ijk}^{s,dp}E_{j}(\omega )E_{k}(\omega ).$$

Additionally, there is a quadrupolar contribution due to the discontinuity of the electric field that originates from a surface layer with a thickness *d* *«* *λ*:

(8) $$P^{s,qp}(2\omega )=\chi_{ijzz}^{s,qp}E_{j}(\omega ){▿}_{z}E_{z}(\omega ),$$

where *z* is the direction orthogonal to the surface. In the bulk only higher order quadrupolar terms contribute to the nonlinear polarization due to its centrosymmetry. They can be described by a fourth rank tensor of the form:

(9) $$P_{i}^{b,qp}(2\omega )=\Gamma_{ijkl}E_{j}(\omega ){▿}_{k}E_{l}(\omega )$$

When comparing the intensities of bulk and surface contributions in non-centrosymmetric media the surface contribution can usually be neglected as the bulk volume is much larger [28]. However, in centrosymmetric media the surface contribution can be dominant, as can be observed at clean reconstructed silicon surfaces in vacuum [29]. For silicon in ambient atmosphere covered by a native oxide, the magnitude of the surface component is reduced but still comparable to the bulk [30]. Hence, the high sensitivity to surfaces makes SHG a suitable tool for the investigation of surfaces and buried interfaces of centrosymmetric material.

**Figure 8:**
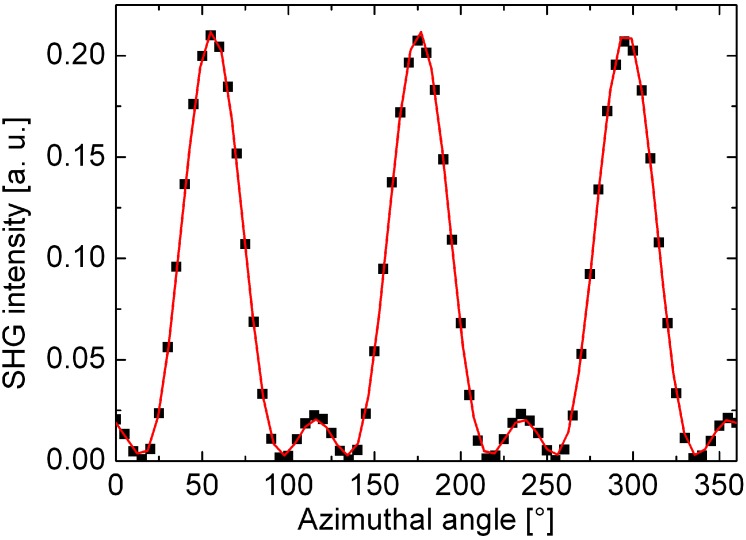
Azimuthal dependence of the second harmonic intensity of a (111) oriented silicon surface measured in reflection. The fundamental and second harmonic beams are p-polarized. The dots represent measured values. The solid line is a least square fit of Equation 17 to the measurement.

When measuring SHG in reflection, the intensity of the detected signal strongly depends on the rotation angle *ϕ* about the surface normal (see Figure 7). The observed oscillatory shape of the signal (Figure 8) has the symmetry of the investigated crystal surface. To be conformal with the symmetry of the crystal the *χ*^(2)^ tensor (Equation 6) has to be invariant under all transformations of the operators of the respective symmetry group. As a consequence many of the $\chi_{ijk}^{\left(2\right)}$ components vanish or become equal to each other to guarantee this invariance [31].

**Figure 9:**
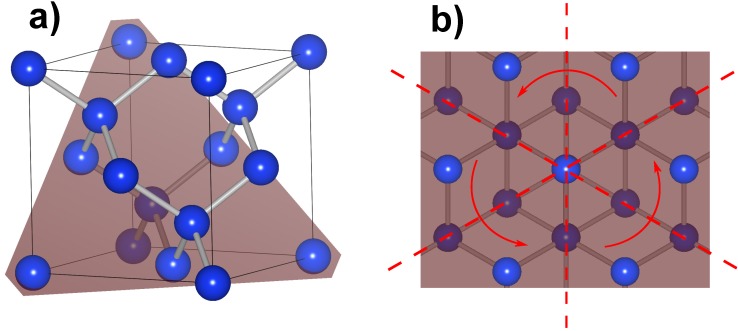
Crystal structure of silicon. (**a**) Unit cell with (111) oriented lattice plane (red); (**b**) Top view of the (111) oriented silicon surface. The symmetry of the surface belongs to group C_3*,v*_ distinguishable from the threefold rotational symmetry.

In the case of (111) oriented silicon the surface belongs to the symmetry group *C*_3*,v*_ with a threefold rotational symmetry (Figure 9). In this case the *χ*^(2)^ has the form [32,33]: (10)
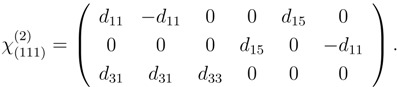


From Equation 10 phenomenological formulas depending on the polarization of the fundamental and second harmonic beam can be derived that describe the intensity modulation observed when the sample is rotated azimuthally [32,33]: 
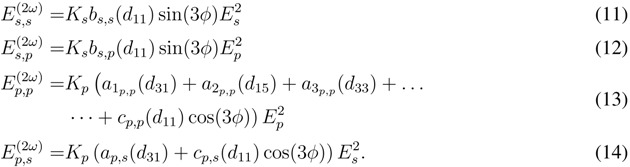


The coefficients *K*_*s/p*_ depend on the angle of incidence Θ and the refractive indices of the medium at frequencies *ω* and 2*ω* , as well as the *a*, *b* and *c*, that depend on the particular tensor elements as well. The s-polarized second harmonic signal shows a sixfold rotational symmetry. Depending on the magnitude of the single tensor components, the p-polarized second harmonic fields mostly show a threefold rotational symmetry as shown in Figure 8. This behavior coincides with the threefold rotational symmetry of the (111) oriented crystal surface as was shown in Figure 9.

Phenomenological expressions for the quadrupolar bulk contribution to the second harmonic signal can be derived similar to the surface contribution and show the symmetry behavior of the surface. Because of their similar symmetries it is generally not possible to distinguish between both contributions unambiguously [28,32,33]. Only in certain cases for (100) and (110) oriented samples both components might be separated, when the phase of the SHG signal is known [34].

The first investigations of the effects of strain on the second harmonic signal in silicon date back in the late eighties [35,36,37], where the inhomogeneous deformation of the crystal lattice at a strained interface layer was investigated in reflection. An increase in the second harmonic signal by more than two orders of magnitude due to mechanical stress was reported by Govorkov *et al.* [5] for a Si_*x*_Ni_*y*_ polycrystalline straining layer. A theory for an analytical estimation of the relation between strain and the induced nonlinear susceptibility was developed by the same authors for diamond-type crystal [5,38] and later with a similar approach by Huang [39]. An alternative phenomenological description, that uses the concept of a strain-dependent photoelastic tensor was presented later by Lyubchanskii *et al.* [40,41].

In the theoretical model developed by Govorkov *et al.* which is referred to as sp^3^-orbital model, the Coulomb interaction between the covalent bonds of the silicon atoms is considered to calculate the second order nonlinear susceptibility. As mentioned before, an important condition for breaking the centrosymmetry of the silicon crystal by strain is its inhomogeneity. This might for example be achieved by a strain gradient parallel to the surface normal created, e.g., by thermal oxidation of a thin silicon oxide layer. By this means a thin strained layer is created in the silicon with a thickness of approximately 5 nm in which the strain decays into the bulk [42,43]. For the sp^3^-orbital model an exponential decay of the strain is assumed as it also occurs at strain induced dislocations [44]. Thus, the strain given by an atomic displacement vector $\overset{\to }{u}$ can be described as:

(15) $$\mathrm{div}\;\overset{\to }{u}=\zeta_{0}\exp(-\Gamma z),$$

where *ζ*_0_ is the deformation value at the surface; Γ is the reciprocal of the deformation characteristic length with Γ*a* *«*1, *a* being the bond length in the crystal. With this assumption Govorkov *et al.* [5] derived a linear relation between the strain induced dipolar second order susceptibility and the stress level *σ*_0_ at the silicon interface [38]:

(16) $$\zeta_{D,def}^{\left(2\right)}\propto \frac{\sigma_{0}}{d}.$$

Recently, Schriever *et al.* [45] confirmed the predicted linear relationship by measuring the reflected SH signal at (111) oriented thermally oxidized silicon interfaces in reflection, using a fundamental wavelength of 800 nm. The 10 to 250 nm thick silicon samples were oxidized under different process conditions (e.g., different temperatures) leading to a variation of the induced film stress and thus to a variation of the strain at the interface between silicon and the cover layer. The p-polarized azimuthal SH signals of a p-polarized fundamental beam (Figure 8) were fitted to the corresponding phenomenological formula (*cf.* Equation 13)

(17) $$I_{pp}^{IH}=|a_{pp}+b_{pp}\cos3\phi |{\;}^{2}I_{p}^{2}.$$

**Figure 10:**
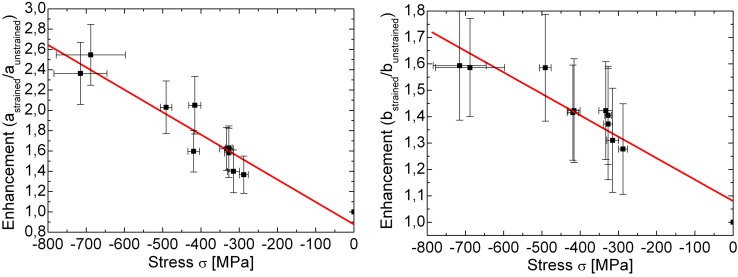
The angular components *a*_*pp*_ (**a**) and *c*_*pp*_ (**b**) of the azimuthal SH susceptibility in Equation 17 versus the stress of the silicon oxide layer on the silicon (111) substrate.

Here, *a*_*pp*_ and *b*_*pp*_ comprise the contributions from surface and bulk electric dipole and electric quadrupole terms. Comparing the enhancement of the both coefficients for different stress values with respect to the components of an unstrained reference sample allows to deduce the dependence of the tensor components on the applied film stress. Figure 10 shows the ratio between the strained and unstrained coefficients *a*_*pp*_ and *b*_*pp*_ revealing the linear relationship.

**Figure 11:**
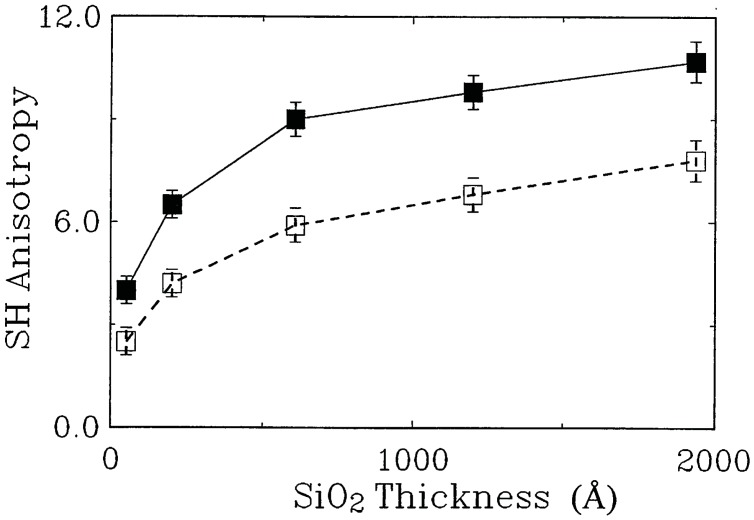
The angular components *c*_*pp*_ (open squares) and *b*_*ss*_ (filled squares) of the azimuthal SH susceptibility in Equation 17 and 11, respectively versus the thickness of the silicon oxide layer on the silicon (111) substrate. Reproduced from Reference [39] with permission from the Japan Society of Applied Physics.

An indirect hint to this relation can also be deduced from Reference [39]. There, the SH signal of a (111) oriented oxidized silicon sample was investigated in dependence of the oxide layer with a thickness of up to 200 nm. As can be seen from Figure 11, the angular coefficients corresponding to the components of *χ*^(2)^ show an exponential dependence on the film thickness. This can be explained with the linearity of Equation 16 in combination with conclusions from Reference [46] that states an exponential dependence between the surface stress on the silicon substrate and the thickness of the thermally grown oxide layer on top.

## 5. Strained Silicon Nonlinear Optical Devices

Besides the fundamental investigation of strain induced second order nonlinearities in silicon, this property was also already employed for photonic devices recently. Initially the major goal consisted in the fabrication of a purely silicon based electro-optic modulator.

**Figure 12:**
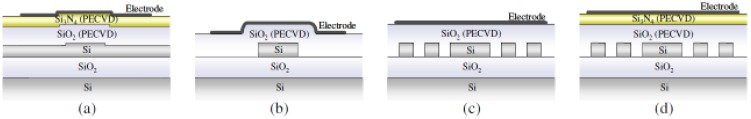
Different waveguide geometries investigated as part of a Mach–Zehnder interferometer. (**a**) shallow etched ridge waveguide; (**b**) fully etched strip waveguide; (**c**) photonic crystal waveguide with PECVD oxide cladding; (**d**) photonic crystal waveguide with combined oxide and nitride cladding. Figure is taken from Reference [47] with courtesy of IEEE.

In an electro-optic modulator the linear electro-optic effect (Pockels-effect) is used to change the refractive index of a material. This index change is connected with the second order nonlinear susceptibility and can be obtained from Equation 6 assuming a time dependent electric field and a slowly varying DC field. In this way the second order susceptibility ensures an electric field dependent susceptibility, which appears as the field dependent refractive index. A common modulator design incorporates a Mach-Zehnder interferometer which consists of two waveguide branches. Applying an electric field to one of the waveguides changes its refractive index and leads to a change of the optical path length for the light wave traveling through this branch. This results in a phase change of the wave at the connection point of the two branches and the interference condition is altered. In this way a relatively small change in refractive index due to the applied voltage can lead to a substantial variation of the exit intensity. To switch from the condition of constructive interference with the maximum intensity at the exit to destructive interference with zero exit intensity a voltage V_*π*_ has to be applied. The aim is to construct energy efficient modulators which have a small V_*π*_, a small footprint and allow high modulations speeds within the GHz range. To employ the strain induced second order susceptibility of silicon in such a Mach-Zehnder geometry, J. Fage-Pedersen *et al.* investigated different waveguide designs applying strained SiO_2_ and Si_3_N_4_ which were both deposited by PECVD [47]. The stress level of the SiO_2_ amounted to a moderate 0.3 GPa while the compressive stress due to the Si_3_N_4_ was a rather large 1 GPa. The modulating voltage was applied in vertical direction between the substrate and an electrode deposited on the top (Figure 12).

Interestingly, a phase modulation could only be observed for the cases shown in Figure 12(b–d) although for the waveguide in (a) also the strongly stressed Si_3_N_4_ layer was present. It seems that the shallowly etched ridge waveguide in (a) can not expand enough to the sides to introduce an inhomogeneous strain across its whole cross section, as the relatively thick continuous Si-device layer on both sides limits the relaxation of the ridge waveguide core. However applying a voltage to a moderately strained SiO_2_ on an etched through strip-waveguide or a photonic crystal waveguide leads to second order nonlinear susceptibilities on the order of *χ*^(2)^ = 5 *−* 10 pm/V. The highest *χ*^(2)^ was obtained for a combination of compressively strained SiO_2_ and Si_3_N_4_-layers on a photonic crystal waveguide.

These *χ*^(2)^-values are approximately connected to the usual electrooptic coefficients via *χ*^(2)^ = *−rn*^4^*/*2, where *n* represents the effective refractive index of the waveguide. However this relation can only be applied to obtain a *χ*^(2)^(*ω,*0) for the special case where one of the involved electric field frequencies is zero (electro-optic effect). It can not be applied to derive an accurate *χ*^(2)^ for second harmonic or difference frequency generation processes from the electro optic coefficient *r*.

**Figure 13:**
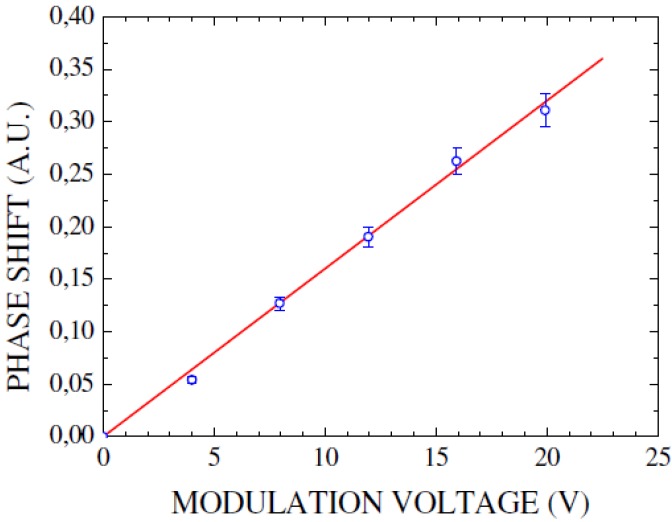
Linear relationship between observed phase shift and modulation voltage indicates the underlying Pockels effect. Figure is taken from Reference [47] with courtesy of IEEE.

The strict linear dependence of the phase shift on the modulation voltage demonstrates that the observed phase shift is indeed caused by the linear electro-optic effect (Figure 13). With the reported values of *χ*^(2)^ realistic modulators still have to have lengths in the cm-range.

**Figure 14:**
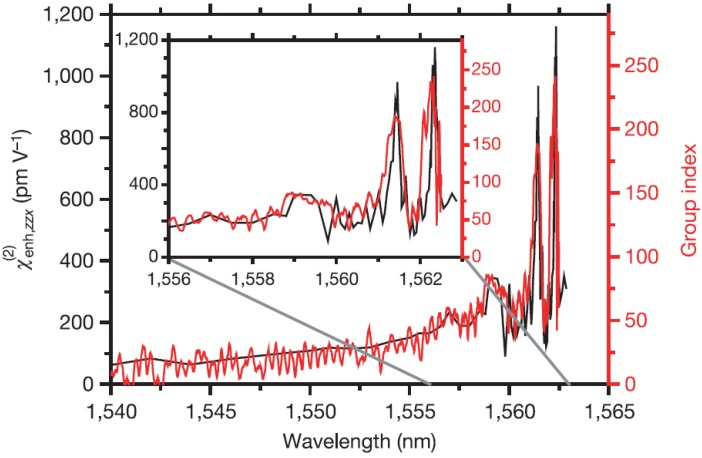
Comparison of the enhanced second order susceptibility $\chi_{enh}^{\left(2\right)}$ and the group index of the photonic crystal waveguide mode. A direct linear relationship is observed and group indices above 100 lead to $\chi_{enh}^{\left(2\right)}$ = 830 pm/V at a wavelength of 1561.5 nm. Figure is taken from Reference [14] with courtesy of Macmillan Publishers Ltd: Nature.

To reduce the size of the interferometer, photonic crystal waveguides, which exhibit a low group velocity, can be employed. These slow light waveguides can lead to a substantial enhancement of nonlinear processes due to the increased group index n_*g*_. This effect was employed successfully by Jacobsen *et al.* [14] to reach an enhanced *χ*^(2)^ of 830 pm/V for a SiO_2_ and Si_3_N_4_ clad photonic crystal waveguide. The comparison of the measured enhanced $\chi_{enh}^{\left(2\right)}$ and the independently determined group index n_*g*_ of the waveguide (Figure 14) demonstrated a direct linear correspondence of these parameters:

(18) $$\chi_{enh}^{\left(2\right)}=\frac{n_{g}}{n}\chi^{\left(2\right)}.$$

Adjusting the period and pore diameter of the photonic crystal, the spectral region in which *n*_*g*_ is high can be shifted to the desired wavelength range, giving the opportunity to build efficient electro-optic modulators with small footprints.

**Figure 15:**
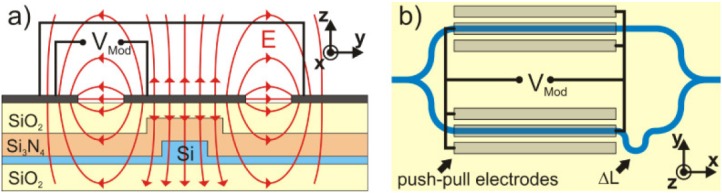
Design of a Mach-Zehnder interferometer applying a push-pull mechanism. (**a**) Electric field at the waveguides. Due to the two gaps between the contacts a vertically directed field at the waveguide is achieved; (**b**) push-pull design ensuring oppositely directed electric fields at both interferometer branches. The figure is taken from Reference [48] with courtesy of the Optical Society of America.

From the measured values of $\chi_{enh}^{\left(2\right)}$ and n_*g*_ the original *χ*^(2)^ of the material due to the inhomogeneous strain is calculated to *χ*^(2)^ = 15 pm*/*V. This corresponds to the value earlier reported for silicon waveguides, which are strained by a Si_3_N_4_ cladding layer. Recently, a further improvement of the inhomogeneously strained silicon Mach-Zehnder interferometer was reported. Chmielak *et al.* [48] demonstrated a Mach-Zehnder interferometer based on a 400 nm wide silicon rib-waveguide which was directly covered with a strained Si_3_N_4_ cladding layer. In addition, a two step annealing process of the structure leads to an increased mechanical stress in the waveguide. To reduce the footprint of the modulator further it was realized in a push-pull geometry where both branches of the modulator are exposed to the modulation voltage with opposite polarity (Figure 15).

This allows to shorten the length of the phase changing contacts in half. Applying a modulation voltage of 30 V a refractive index change of Δ*n*_*Si*_ = 2*.*4 *×* 10^*−*5^ was achieved. This corresponds to a *χ*^(2)^ = 122 pm*/*V within the rib waveguide. Increasing the width of the waveguide from 400 nm to 450 nm leads to a decrease of *χ*^(2)^ to 71.5 pm/V. This shows that the inhomogeneous strain distribution within the waveguide is important and that the areas around the side facets of the waveguides contribute most to the *χ*^(2)^ of the waveguide. The possibility to locally induce a second order nonlinear susceptibility in silicon due to an inhomogeneous strain extends the use of silicon in the realm of active integrated optical elements. With a maximum *χ*^(2)^ = 122 pm*/*V the corresponding electro-optic coefficient is comparable to the one of KDP. Moreover the refractive index change, which is crucial for the electro-optic modulation, is almost an order of magnitude higher in the silicon waveguides than in KDP due to the high refractive index of silicon. The demonstrated modulators, which were mainly proof of concept devices, were only modulated with frequencies up to 500 kHz. However the quasi-instantaneous Pockels effect should allow much larger modulation frequencies. Furthermore the charge carrier concentration within the silicon is not changed, so that energy intensive loading and unloading of the waveguide with charge carriers (current flow!) can be avoided. In usual silicon modulators a change in refractive index is achieved by flooding or emptying the waveguide with carriers using the dependence of the refractive index on free carrier concentration for modulation.

Recently, the interest in strain induced second order nonlinearities in silicon went beyond the field of electro-optic modulation and focused on light frequency transformation. Hon *et al.* investigated theoretically frequency conversion processes in channel waveguides with periodically deposited tensile and compressive stressed silicon nitride films [49]. Such a periodic modulation that can be used to create quasi-phasematching could be used to further enhance second harmonic processes as it has been investigated recently by Cazzanelli *et al.*, who have investigated this effect in inhomogeneously strained silicon waveguides [50]. A Si_3_N_4_ covered SOI strip waveguide was used to achieve local strains of up to 0.25% and a strong strain gradient. The pump wavelength was chosen between 2050 nm and 2300 nm so that the energy of the second harmonic was just below the interband absorption of silicon. As no phase matching efforts were undertaken the intensity of the generated second harmonic light varies periodically along the waveguide, so that the observed second harmonic intensity at the waveguide exit depends crucially on the length of the waveguide. Nevertheless a conservative lower limit of *χ*^(2)^ = 40 pm*/*V can be deduced from the measurements when it is assumed that the waveguide is just cut at a length of maximum second harmonic intensity.

## 6. Conclusions

Although the field of strained silicon nonlinear photonics is still in its infancy, the interest has increased strongly. The presented examples show intriguingly how local strain engineering can change material properties in a qualitative way. The recently published works will certainly inspire further research into active silicon photonic devices. Possibly, the parameter strain can enhance the spectrum of silicon photonic devices and fuel this field of work in a similar way as it has done in the field of microelectronics and microelectromechanics.
